# Moderately hypofractionated versus conventionally fractionated radiation therapy with temozolomide for young and fit patients with glioblastoma: an institutional experience and meta-analysis of literature

**DOI:** 10.1007/s11060-022-04151-z

**Published:** 2022-11-10

**Authors:** Phoebe Chidley, Mihir Shanker, Claire Phillips, Neda Haghighi, Mark B. Pinkham, James R. Whittle, Joseph Sia

**Affiliations:** 1 Department of Radiation Oncology, Christchurch Hospital, Christchurch, Canterbury 8011 New Zealand; 2 Department of Radiation Oncology, Princess Alexandra Hospital, Brisbane, QLD 4102 Australia; 3 University of Queensland, Brisbane, QLD 4072 Australia; 4 Department of Radiation Oncology, Peter MacCallum Cancer Centre, Melbourne, VIC 3000 Australia; 5 Epworth Radiation Oncology and Icon Group, Melbourne, VIC 3000 Australia; 6 Department of Medical Oncology, Peter MacCallum Cancer Centre, Melbourne, VIC 3000 Australia; 7 Personalised Oncology Division, The Walter and Eliza Hall Institute of Medical Research, Parkville, VIC 3052 Australia; 8 Department of Medical Biology, University of Melbourne, Parkville, VIC 3010 Australia; 9 Sir Peter MacCallum Department of Oncology, The University of Melbourne, Parkville, VIC 3010 Australia

**Keywords:** Glioblastoma, Radiotherapy, Hypofractionation, Temozolomide, Overall survival

## Abstract

**Purpose:**

Shorter hypofractionated radiation therapy (HF-RT) schedules may have radiobiological, patient convenience and healthcare resource advantages over conventionally fractionated radiation therapy (CF-RT) in glioblastoma (GBM). We report outcomes of young, fit GBM patients treated with HF-RT and CF-RT during the COVID-19 pandemic, and a meta-analysis of HF-RT literature in this patient subgroup.

**Methods:**

Hospital records of patients with IDH-wildtype GBM treated with HF-RT (50 Gy/20 fractions) and CF-RT (60 Gy/30 fractions) between January 2020 and September 2021 were reviewed. Overall survival (OS) and progression-free survival (PFS) were estimated using the Kaplan-Meier method. Univariable analysis was performed using Cox regression analysis. A systematic search and meta-analysis of studies from January 2000 to January 2022 was performed.

**Results:**

41 patients were treated (HF-RT:15, CF-RT:26). For both HF-RT and CF-RT groups, median age was 58 years and 80–90% were ECOG 0–1. There were more methylated tumours in the HF-RT group. All patients received concurrent/adjuvant temozolomide. At 19.2 months median follow-up, median OS was 19.8 months and not-reached for HF-RT and CF-RT (p = 0.5), and median PFS was 7.7 and 5.8 months, respectively (p = 0.8). HF-RT or CF-RT did not influence OS/PFS on univariable analysis. Grade 3 radionecrosis rate was 6.7% and 7.7%, respectively. 15 of 1135 studies screened from a systematic search were eligible for meta-analysis. For studies involving temozolomide, pooled median OS and PFS with HF-RT were 17.5 and 9.9 months (927 and 862 patients). Studies using shortened HF-RT schedules reported 0–2% Grade 3 radionecrosis rates.

**Conclusion:**

HF-RT may offer equivalent outcomes and reduce treatment burden compared to CF-RT in young, fit GBM patients.

**Supplementary Information:**

The online version contains supplementary material available at 10.1007/s11060-022-04151-z.

## Introduction

Glioblastoma (GBM) is a uniformly fatal illness with a poor prognosis [1]. Trimodality treatment for GBM involves maximal safe resection followed by radiation therapy (RT) and temozolomide chemotherapy. The standard-of-care 6-week conventionally-fractionated RT (CF-RT) schedule of 60 Gy in 30 daily fractions for GBM was established by the MRC BR2 dose-escalation study in 1991 [2] and further reinforced by the landmark EORTC-NCIC trial in 2005 [3], which added concurrent and adjuvant temozolomide to this schedule and demonstrated an overall survival (OS) improvement from 2% to 10% at 5 years. Disappointingly, there has not been significant progress in the state of first-line adjuvant therapy since then, with many treatment-intensification strategies including radiation dose-escalation and the addition of other chemotherapeutic and targeted agents showing no additional survival benefit [4–7].

In RT, hypofractionation is the delivery of fewer treatment fractions by using larger doses per fraction compared to conventional fractionation (the latter usually defined as 1.8–2.0 Gy per fraction). The safe delivery of hypofractionated-RT (HF-RT) is made possible with modern RT techniques, which can shape radiation beam fluences to conform to target volumes tightly and therefore spare adjacent normal tissue. Mathematical modelling of GBM suggests that mildly accelerated and hypofractionated-RT may partially counteract the rapid repopulation rate of GBM [8], leading to radiobiological advantages in tumour control. However, another important benefit of shorter HF-RT schedules is the reduction of treatment burden for the patient, in the context of a devastating diagnosis with significant physical and psychosocial sequelae as well as a distressing prognosis. In GBM, HF-RT is routinely offered to elderly (> 65–70 years) and/or poor performance status patients, following a series of randomised trials that demonstrated non-inferiority of these schedules compared to CF-RT [9–11]. However, for younger and fit patients, HF-RT has not been well investigated in the temozolomide era.

The community risks and healthcare system challenges posed by the coronavirus-2019 (COVID-19) pandemic has forced re-evaluation of clinical practice internationally, including brain tumour management [12–15]. To minimise hospital footfall, manage staff shortages and ensure completion of patient treatment during the virus outbreaks in 2020–2021, COVID-19 protocols for RT were introduced in our institution during periods of high community viral spread. This includes using a standardised protocol of a 4-week HF-RT schedule for young, fit adult GBM patients, instead of the 6-week CF-RT schedule. Herein, we compare outcomes between patients treated with HF-RT and CF-RT. The COVID-19 protocol was enacted intermittently over 2020–2021, and therefore this study represents a comparison of two contemporaneously-treated cohorts comprising the same patient population in a single institution. To present our findings in context, we also performed an updated systematic review and meta-analysis of the current literature on the use of HF-RT in young, fit patients with GBM.

## Methods

### Patients

Hospital records of consecutive patients with WHO Grade 4 GBM, isocitrate dehydrogenase (IDH)-wildtype (as per the WHO 5th edition classification [16]) who received 50 Gy in 20 fractions (HF-RT) or 60 Gy in 30 fractions (CF-RT) at the Peter MacCallum Cancer Centre, Melbourne, Australia (PMCC) between 1 and 2020 and 30 September 2021 were retrospectively reviewed. HF-RT was used in lieu of CF-RT when hospital COVID-19 protocols were enacted during periods of high community virus spread. In lower-risk periods, this RT schedule modification was relaxed with resumption of CF-RT use.

All patients underwent magnetic resonance imaging (MRI) with advanced sequences of the brain [17] pre- and post-maximal safe resection and were discussed in the institutional neuro-oncology multidisciplinary team (MDT) meeting. Performance status was graded according to the Eastern Cooperative Oncology Group (ECOG) system. All tumours were tested for IDH1 R132H mutation by immunohistochemistry. For patients under the age of 55 years in whose tumours were IDH1 R132H immunonegative, variants in IDH1 codon 132 or IDH2 codon 7 were further assessed by next generation sequencing or pyrosequencing. Unfortunately, O^6^-methylguanine-DNA-methyltransferase (MGMT) promoter methylation status was not consistently tested during the period of this study. Due to ethical reasons, retrospective MGMT testing could only be performed on patients who were still alive at the time of analysis. Approval by the institutional ethics committee to conduct this study was obtained.

## Radiation therapy, chemotherapy and follow-up

Patients underwent a RT planning computed tomography (CT) with 1 mm slices, using a thermoplastic mask for immobilisation. The planning scan was fused with pre- and post-operative MRI T1 with gadolinium and T2/FLAIR sequences. A single dose-level approach was adopted for target delineation, whereby the gross tumour volume (GTV) encompassed the surgical cavity and any residual contrast-enhancing and non-enhancing T2/FLAIR-hyperintense tumour. Clinical Target Volume (CTV) expansion margin was 1.5 cm, with provision to reduce to 1 cm for large tumours per clinician judgement, respecting anatomical boundaries. The Planning Target Volume (PTV) expansion was 0.3 cm. All patients were planned and treated using a volumetric-modulated arc therapy (VMAT) technique. Major organs-at-risks included the brainstem, optic apparatus, cochlea, and lens. Dose constraints were adapted from consensus guidelines [18]. Specific dose constraints for the HF-RT schedule were point maximum dose < 50 Gy to the brainstem and optic apparatus.

All patients received concurrent temozolomide (75 mg/m^2^ daily, orally) and adjuvant temozolomide (150–200 mg/m^2^ for 5 days every 28 days, orally) for 6 months or until disease progression if earlier. Patients were reviewed weekly during RT to monitor for toxicity. Acute toxicity was scored using the Common Terminology Criteria for Adverse Events (CTCAE) v5.0 and included toxicity experienced up to 30 days post completion of RT. Due to the retrospective nature of this study, only Grade ≥ 2 toxicities could be reliably reported. Standard follow-up post completion of RT included a phone review at 1 week, a clinical assessment and MRI brain at 1 month, then at 3-monthly intervals or guided by clinical symptoms. Definition of radiological progression was based on clinical and radiological findings. Cases were discussed at MDT for consensus and, where radiological findings were indeterminate, short interval imaging was undertaken prior to deciding outcome.

### Statistical analysis

Statistical comparison between groups was performed using the t and chi-square tests for continuous and categorical variables, respectively. Median follow-up time was assessed by the reverse Kaplan-Meier method. OS, defined as time from surgery to death, and progression free survival (PFS), defined as time from surgery to radiological progression or death, were assessed using the Kaplan-Meier method. Survival comparison between groups was performed using the log-rank method. Univariable analysis was undertaken using Cox regression analysis to assess the impact of covariates on OS and PFS. Due to the limited number of patients, multivariable analysis was not performed.

## Systematic review and meta-analysis

**Fig. 1:**
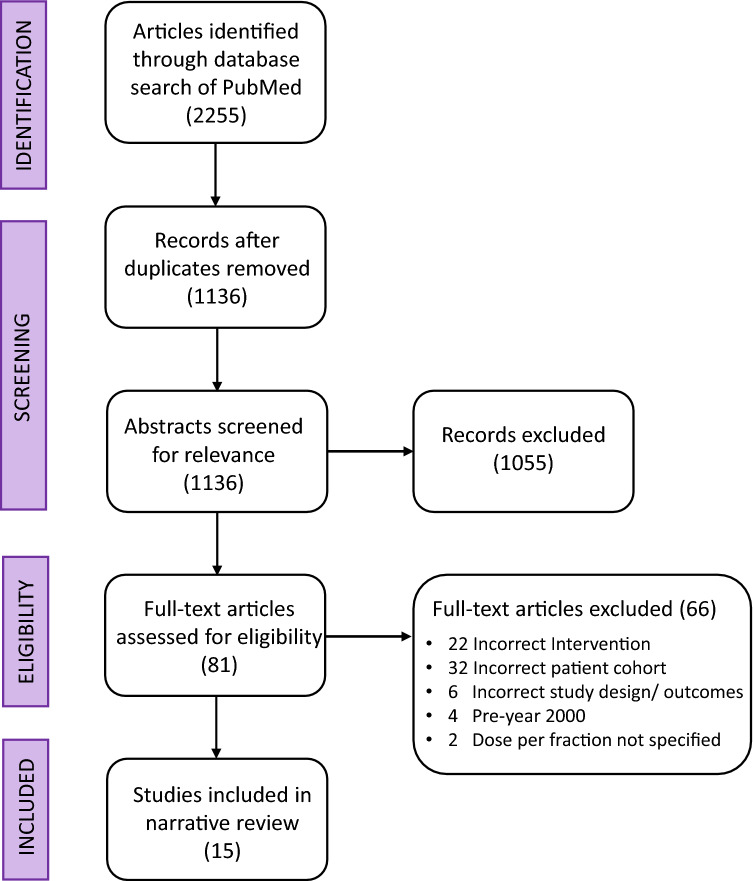
PRISMA diagram for systematic search

A literature search was undertaken of the PubMed database for articles published between 1 and 2000 to 31 January 2022. The search terms were “Glioblastoma”[Title] AND (radiotherapy[Title] OR irradiation[Title] OR radiation[Title]) NOT Review[Publication Type] NOT “Meta-Analysis”[Publication Type] NOT “Systematic Review”[Publication Type] NOT mice[MeSH Terms] NOT pre-clinical[MeSH Terms] AND English[Language]”. To be included for review, studies had to (1) involve patients with GBM or high grade glioma in the *de novo* setting, (2) have a patient cohort of median age < 65 years and good performance status (defined as ECOG 0–2, Karnofsky performance score ≥ 70, or NRG-GBM-RPA [19] Classes I and II), (3) used a moderate HF-RT schedule (defined as > 2 Gy/fraction and ≤ 5Gy/fraction) with the radiation dose-fractionation specified; and (4) report OS and/or PFS. Two authors performed the study screening (PC and JS). The Covidence software (Veritas Health Innovation, Melbourne, Australia) was used to import the PubMed search findings and perform abstract and full text review. A Preferred Reporting Items for Systematic Reviews and Meta-Analyses (PRISMA) diagram (Fig. 1) was generated.

Median OS and PFS for each outcome of interest were extracted from the analysed articles. Individual study median metrics and standard deviations for sample means were calculated using the population standard deviation and sample size in combination with the generic inverse variance method for the fixed effect model. As included studies were performed at independent facilities with independent population groups, a DerSimonian and Laird random effects model [20] was additionally used to assess pooled results. Heterogeneity was assessed using Cochranes Q test with a p < 0.1. Tau squared and Tau were additionally used to assess inter-study variance for random effects models. Studies with missing data that were not imputable were excluded from individual models as appropriate. All statistical analysis was performed using Comprehensive Meta-Analysis software version 3 (Biostat Inc., Englewood, NJ, USA) and MedCalc (MedCalc Software Ltd, Belgium).

## Results

### Patient and tumour characteristics

41 consecutive patients were treated and met inclusion criteria during the study period. 15 patients received HF-RT and 26 patients received CF-RT. Patient and tumour characteristics are outlined in Table 1. The median ages in both groups were similar at 58 years (p = 0.96), with the majority (80–90%) being ECOG 0–1 in performance status (p = 0.51). 20% of patients in the HF-RT group underwent tumour biopsy only, compared to 11.5% in the CF-RT group (p = 0.76). The proportion of patients with confirmed methylated MGMT status was higher in the HF-RT group (p = 0.01). All patients received concurrent and adjuvant temozolomide.

**Table 1:** Patient and tumour characteristics

	HF-RT (50 Gy/20 fractions)	CF-RT (60 Gy/30 fractions)	p-value
n	15	26	
Age (median [IQR])	58 [51,60.5]	58.5 [50.3–62.8]	0.957
Sex (%)			0.570
M	10 (66.7)	15 (57.7)	
F	5 (33.3)	11 (42.3)	
ECOG PS (%)			0.510
0	4 (26.7)	8 (30.8)	
1	8 (53.3)	16 (61.5)	
2	3 (20.0)	2 (7.7)	
Largest dimension, mm (mean [SD])	38.9 (19.1)	43.2 (18.0)	0.475
Extent of resection			0.756
Biopsy	3 (20.0)	3 (11.5)	
STR	6 (40.0)	12 (46.2)	
GTR	6 (40.0)	11 (42.3)	
Time to RT, days (mean [SD])	33.1 (8.5)	31.8 (7.1)	0.584
MGMT status (%)			0.011
Methylated	6 (40.0)	4 (15.4)	
Unmethylated	2 (13.3)	16 (61.5)	
Unknown	7 (46.7)	6 (23.1)	
Ki67 (mean [SD])	28.8 (12.5)	29.3 (14.0)	0.913

*IQR* Interquartile range, *ECOG* Eastern Cooperative Oncology Group, *PS* Performance status, *SD* Standard deviation

## Overall and progression-free survival

**Fig. 2:**
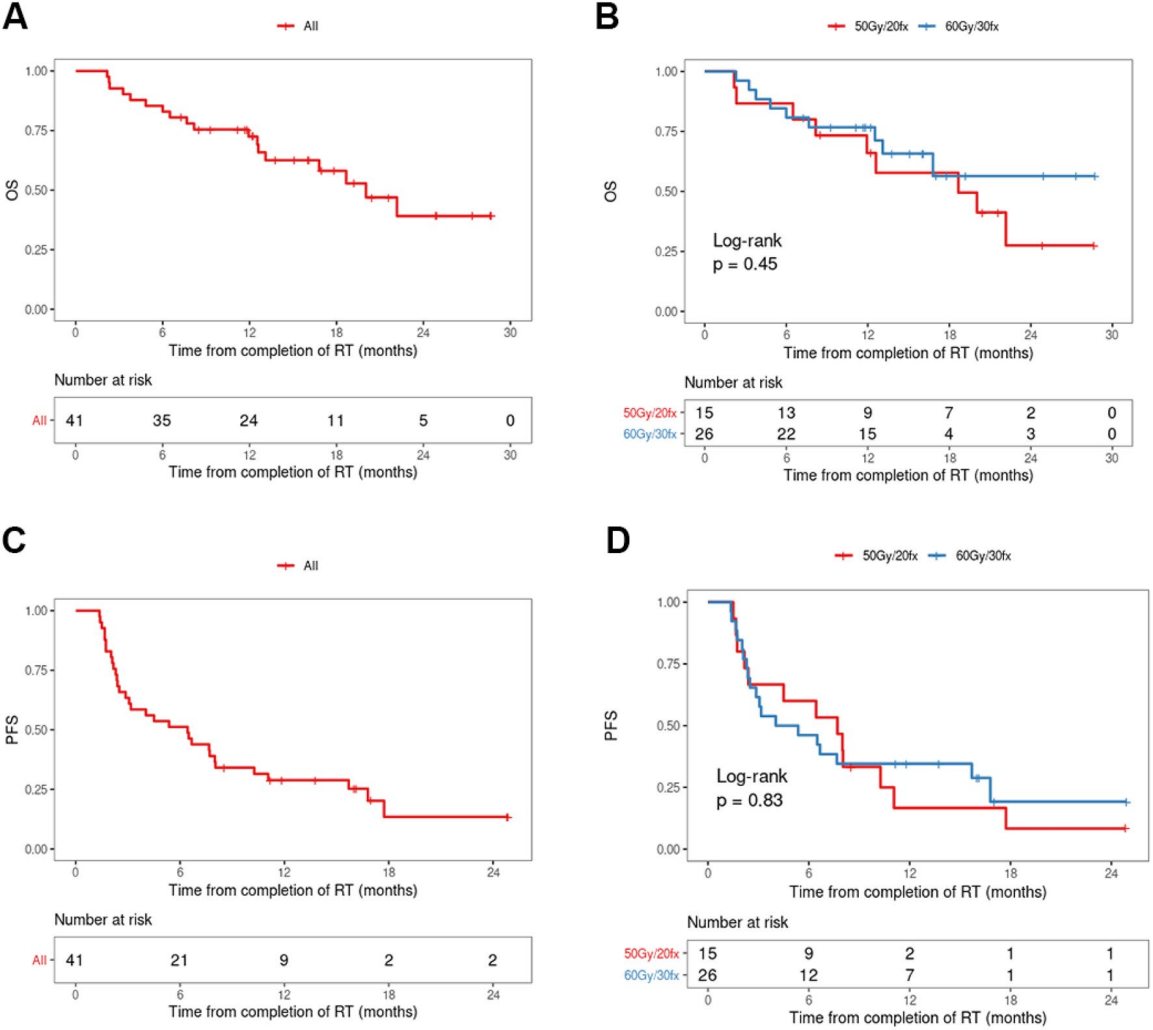
Overall survival (**A**, **B**) and progression-free survival (**C, D**)

With an actuarial median follow-up of 19.2 months, median OS for all patients was 21.5 months. The median survival for the HF-RT group was 19.8 months, compared to not reached for the CF-RT group (p = 0.5, Fig. 2 A, B). The median PFS for all patients was 7.5 months with no statistically significant difference between the two groups (7.7 and 5.8 months for HF-RT and CF-RT, respectively, p = 0.71) (Fig. 2 C, D*)*.

Age, sex, performance status, Ki67 index, extent of resection, time from surgery to RT, MGMT methylation status, and RT schedule (CF-RT versus HF-RT) were analysed for prognostic correlation with OS and PFS in univariable analyses. Age (HR 1.06 per year, p = 0.04) and time from surgery to RT (HR 1.07 per day, p = 0.02) were the only factors to have a significant p-value for association with OS. The use of either HF-RT or CF-RT did not influence OS or PFS.

## Acute toxicity and radionecrosis

Three patients (11.5%) in the CF-RT group had treatment-related Grade 2 fatigue in the second half of the chemo-RT course, compared to no documented cases in the HF-RT group. One case (6.7%) of Grade 2 nausea was noted in the HF-RT group, requiring initiation of additional anti-emetics. All patients in both groups completed treatment. Four patients developed radionecrosis in each cohort (interchangeably labelled pseudoprogression in the literature), representing 27% and 15% (p = 0.38) with a median onset at 5.8 and 7.4 months (p = 0.58) in the HF-RT and CF-RT groups, respectively. In the HF-RT cohort, 1 patient (6.7%) had Grade 3 radionecrosis (symptomatic, requiring surgery), while the other 3 patients remained asymptomatic (radiological diagnosis). In the CF-RT cohort, 2 patients (7.7%) underwent surgery for radionecrosis. Of those who developed radionecrosis in the HF-RT group, 2 had a methylated MGMT promoter status, 1 unmethylated, and 1 unknown. For the CF-RT group, 2 were methylated and 2 were unmethylated.

## Systematic review and meta-analysis of literature

**Fig. 3:**
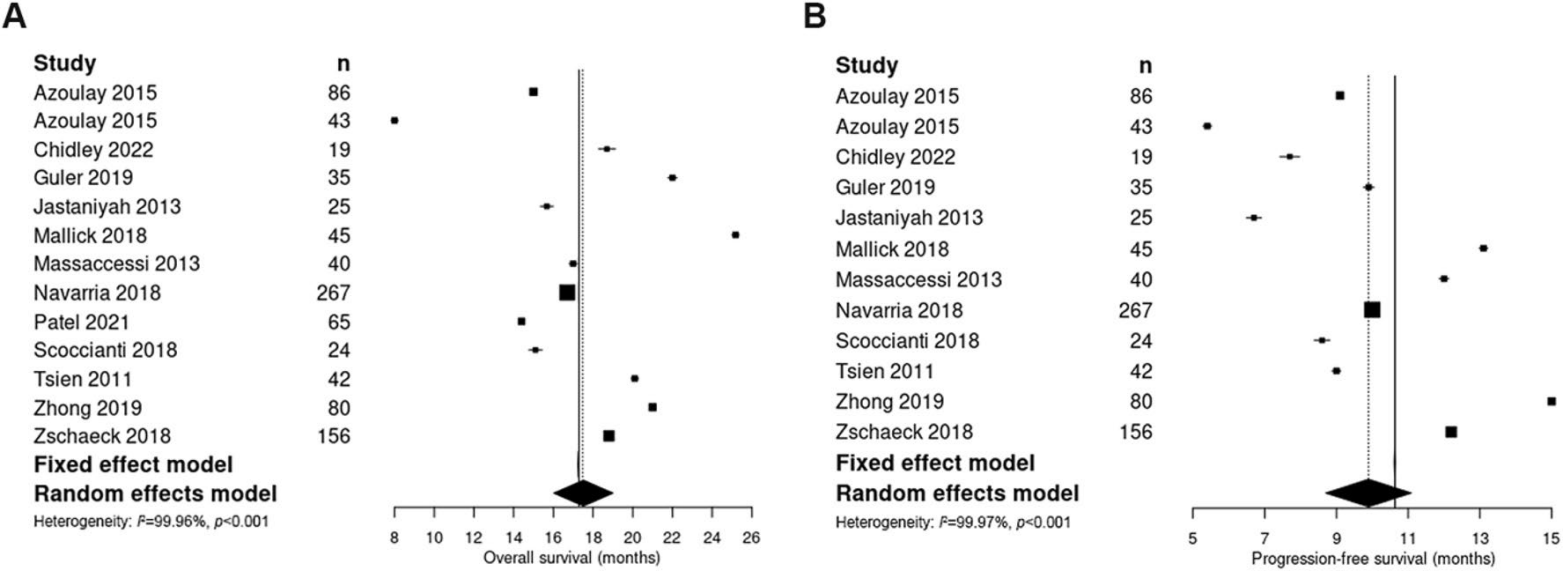
Individual and pooled OS (**A**) and PFS (**B**) from studies of HF-RT in young and fit patients with GBM

A total of 1136 articles were identified for screening by systematic search. 15 studies involved a majority of younger and good PS patients (defined by criteria 2 in Methods) and thus were eligible for final analysis (Fig. 1). These studies are summarised in Table 2. The 32 other studies that included or focused on elderly and poor performance status patients are summarised in *Supplementary Table 1*. In studies conducted in the temozolomide era (12 studies assessed, including an abstract and the present study), the pooled median OS for HF-RT (927 patients) was 17.5 months (95% CI 16.0–19.0), and the pooled median PFS (862 patients) was 9.9 months (95% CI 8.7–11.1) (Fig. 3).

**Table 2:** Clinical studies of moderate HF-RT in GBM with a majority of young and fit patients

Reference	Study design	n	Patient characteristics		Arms			Outcomes		Toxicity
			Median age (years)	Performance status	Dose fractionation	Dose levels	TMZ use	Median OS (months)	Median PFS (months)	
*Group 1: Prospective studies that examined a radiation boost on a CF-RT backbone over 5-6 weeks*										
Massaccesi 2013 [21]	Prospective	40	54	72.5% ECOG 0-1 22.5% ECOG 2 5% ECOG 3	Dose escalation of PTV1 to 60Gy, 62.5Gy, 65Gy, 67.5Gy, 70Gy in 25#	Two	Yes	17.0 (2yOS: 21.9%)	12.0 (2yPFS: 0%)	3 of 14 pts (21%) with dose-limiting toxicity with 70Gy/25# (1 pt died in setting of haematological toxicity; 2 pts Gr3 neurological toxicity - both with seizures, 1 with aphasia). Late: 5 of total 40 pts (12.5%) neurological toxicity (5 pts headaches, ungraded [2 ‘mild’, 3 ‘moderate’], 1 pt re-operated showing tumour recurrence and RN). Dose level received ND.
Monjazeb 2012 [22]	Phase I prospective	21	Mean age 55	Eligibility: KPS >70	Dose-escalation to boost target volume to 70Gy, 75Gy and 80Gy (initial target volume 50.4Gy/28#)	Two	No	13.6 (range 0.9-40.2) (2yOS: 19%) No difference in OS or PFS between groups	6.5 (range 0.9-40.2)	No dose-limiting toxicities found. Acute: 8 pts Gr3 (2 pts reversible otitis media attributed to RT; other toxicities attributed to disease progression or steroids, included 2 pts DVT, 1 pt GI toxicity, 4 pts neurotoxicity [1 of these pts with Gr4 hyperglycaemia]). Late: Nil Gr4 tox, 2 pts (9.5%) Gr3 (1 pt DVT, 1 pt unspecified neurotoxicity). 11 pts required re-resection, none found to have RN alone - all in conjunction with recurrence)
Tsien 2011 [23]	Prospective	42	56	78.6% KPS 90-100	Dose escalation to boost volume to total dose 66-81Gy/30#	Two	Yes	20.1 (95% CI 14-32.5)	9.0 (95% CI 6-11.7)	Gr3+ late neurological toxicity in 2 of 7 pts (29%) with 78Gy, and 1 of 9 pts (11%) with 81Gy. No RN demonstrated with dose < 75Gy. Gr3 late otitis in 1 pt (dose level received ND).
*Group 2: Prospective studies that shortened overall treatment time to below 5 weeks*										
Jastaniyah 2013 [24]	Phase 1 dose escalation	25	53	Eligibility: KPS > 70	Dose escalation from 54.4Gy/20# to 60Gy/22#	One	Yes (76%)	15.7 (95% CI 11.5-20)	6.7 (95% CI 4.0-14.0)	No dose limiting toxicity. 2 cases of Gr3-4 haematological toxicity and 1 case of Gr4 infection.
Mallick 2018 [25]	Phase 2 randomised	43	45	Eligibility: KPS >70	60Gy/30#	Two	Yes	18.1 (95% CI 14.52-NR)	14.1 (95% CI 10.5-15.9)	Acute: 1 pt DVT Late: No cases of RN recorded
		46			'HART' (SIB 60Gy/20# high-risk PTV and 50Gy/20# low-risk PTV)	Two	Yes	25.2 (95% CI 12.89-NR) [P = 0.3]	13.1 (95% CI 10-NR)	Acute: 2 pts hospitalised with features raised ICP, 2 pts steroid requirement post RT, 1 pt RT interruption, 1 pt DVT, 4 pts Gr3-4 thrombocytopaenia Late: 1 case (2.2%) of RN, ungraded
Scoccianti 2018 [26]	Phase 2 multicentre	24	61	46% KPS 90-100, 54% KPS 70-80	Boost dose of 67.5Gy/15# (52.5Gy/15# non-boost volume)	Two	Yes	15.1	8.6	1 case (2.4%) Gr4 RN (requiring surgical resection)
*Group 3: Comparative retrospective studies*										
Azoulay 2015 [28]	Retrospective	147	59	95% KPS >70	60Gy/30#	One	Yes (95.2%)	16.0	9.2	ND
		86	57	88% KPS >70	60Gy/20#	One	Yes (98.8%)	15.0 9.1	9.1	ND
		43	72	54% KPS >70	40Gy/15#	One	Yes (55.8%)	8.0	5.4	ND
Guler 2019 [29]	Retrospective	91	54	60.4% KPS 90-100, 39.6% KPS 70-80	60Gy/30#	One	Yes	14.9 (95% CI 10.6- 19.2)	Local PFS: 9.9	ND
		35	57	57.1% KPS 90-100, 42.9% KPS 70-80	70Gy/30#	Two	Yes	22.0 (95% CI 13-31) [*P*=0.45]	Local PFS: 8.9 (*P*=0.89)	ND
Navarria 2018 [30]	Retrospective propensity score matched analysis	98	61	100% KPS >70	60Gy/15#	Two	Yes	16.7 (95% CI 14.5-18.9) 2yOS: 33.3%(+ 5.4%)	10.0 (95% CI 8.2-11.8)	0% pts discontinued RT due to disease progression. 2 pts (2%) transient neurological deterioration (partial seizure, aphasia). Gr1-2 RN 20%. Gr3-4 RN 0%.
		169	61	97% KPS > 70	60Gy/30#	ND	Yes	17.9 (95% CI 16.0-19.9); 2yOS: 32.7%(+ 5.2%)	12.3 (95% CI 8.7–15.9)	9% pts discontinued RT due to disease progression. Gr1-2 RN 0%. Gr3-4 RN 0%.
*Group 4: Other studies*										
Floyd 2004 [31]	Prospective	20	60	Eligibility: KPS >70	50Gy/10# to enhancing tumour, 30Gy/10# to oedema	Two	No	7.0 (range 1–23)	6.0 (range 0–12)	3 pts (20% of 15 pts evaluable) with Gr4 RN requiring surgical excision
Phillips 2003 [32]	Prospective randomised	36	59	83% ECOG 0-1	60Gy/30#	One	No	10.3 (CI 7.8-14)		No significant late toxicity (per protocol definition) reported.
		32	58	91% ECOG 0-1	35Gy in 10#	One	No	8.7 (CI 7.4 - 10.7) [*P* = 0.37]		
Sultanem 2004 [33]	Prospective	25	55	88% KPS > 70	Concomitant boost (GTV receiving 60/20#, PTV 40Gy/20#)	Two	No	9.5 (2.8-22.9)	5.2 (range 1.9-12.8)	1 pt vision loss, unlikely unrelated to RT (developed 9 months post RT; optic chiasm received 40Gy at 2Gy/fraction)
Usman 2015 [34]	Retrospective	62	50	76% ECOG 0, 24% ECOG 1	48Gy/16#	One	No	9.0		ND
Zhong 2019 [35]	Retrospective	80	50	71.2% KPS >80	Concomitant boost up to 64Gy/27#	Two	Yes	21.0 (95%CI: 17.5-24.4). 2y OS 41.6%	15.0 (95%CI: 11.0-18.9). 2yPFS 27.6%	3 pts (3.7%) RN, with 2 pts undergoing surgery for symptomatic enhancing tissue change. Cognitive disturbance (late) 4 pts (5%), ungraded.
Zschaeck 2018 [36]	Retrospective	23	51	61% RPA Class 1-2	SIB technique to total 66Gy/30#	Two	Yes (78.3% received concurrent)	18.8 (range 5-37.8)	12.2	Acute: Nil Gr3+ toxicity recorded. Late: Asymptomatic RN 1 pt
		133	62	66% RPA Class 1-2	60Gy group (60Gy/30# or 59.2Gy/37# BD)	Two	Yes (97% received concurrent)	15.3 (range 2-48.1) [*P* = 0.012]	7.6 (*P* = 0.011)	ND

*ND* Not described,* RN* Radionecrosis,* Pt* patient,* Gy* Gray,* #* fractions,* y* years,* SIB* Simulated integrated boost,* TMZ* Temozolomide,* KPS* Karnofsky Performance Status,* RPA* Recursive Partitioning Analysis,* Gr* Grade of toxicity,* ICP* Intracranial pressure,* DVT* Deep vein thrombosis

Overall, studies can be categorised into (1) prospective trials that examined radiation dose-escalation using a simultaneous-integrated boost (with a greater dose per fraction) to a high-risk volume, on the backbone of a CF-RT schedule delivered to a larger, low-risk volume over 5–6 weeks, (2) prospective trials that reduced the overall treatment time to < 5 weeks, (3) comparative retrospective studies of HF-RT versus CF-RT, and (4) other studies. The first group consists of three Phase I dose-escalation studies, in which radiation boosts to total doses of 60–81 Gy were tested over the CF-RT backbone (or similar) by employing larger fraction sizes [21–23]. Rates of Grade ≥ 3 neurological toxicity and radionecrosis extended up to 29%, but Tsien et al. found that radionecrosis was only demonstrated with total doses of > 75 Gy [23]. In contrast, radionecrosis rates were only 0–2.4% in the second group of studies [24–26], which used HF-RT to shorten the RT course rather than primarily as a dose-escalation strategy, as is relevant to the present study. Interestingly, both randomised trials by Mallick et al. [25] and Patel et al. [27] (the latter in abstract form only, thus not included in Table 2) found a trend towards OS benefit with HF-RT compared to CF-RT (25.2 versus 18.1 months, and 14.4 versus 11.1 months, respectively). Three retrospective comparative studies of HF-RT versus CF-RT comprised the third group [28–30]. A notable study is a propensity-matched analysis by Navarria et al. on patients who received 60 Gy in 15 fractions or CF-RT with temozolomide [30]. While OS was not different between the two groups, no patients in the HF-RT group had to discontinue RT due to disease progression, compared to 9% in the CF-RT group (similar to the 10% reported in the NCIC-EORTC trial which used CF-RT [3]). However, Grade 1–2 radionecrosis was seen more frequently with HF-RT compared to CF-RT (20% versus 0%). The fourth group of studies [31–36], consisting of single-arm retrospective reports as well as older studies that used suboptimal doses and/or lack of concurrent chemotherapy, should be interpreted with caution and are not further described here.

## Discussion

In this study, we report our experience of using a 4-week HF-RT schedule (50 Gy in 20 fractions) in young, fit patients with GBM, as necessitated by the COVID-19 pandemic. Importantly, we contrast this with a contemporaneous, rather than historical, cohort of patients who received the standard 6-week CF-RT schedule (60 Gy in 30 fractions) at the same institution and under the care of the same treating clinicians, thus offering a more reliable non-randomised comparison. We observed no statistically significant differences in OS or PFS between the HF-RT and CF-RT groups. The clear limitation of our study is the small number of patients and apparent imbalance in MGMT methylation status between groups. Nonetheless, the true nature of the latter is obscured by a 32% rate of unknown methylation status across the entire cohort. Unfortunately, due to ethical reasons, we were only able to retrospectively perform MGMT methylation testing for patients who are still alive at the time of analysis. Even so, considering the data and review of literature presented herein, we maintain that there is sufficient impetus to test the use of HF-RT in young and fit patients with GBM in appropriately powered, randomised studies.

Both HF-RT and CF-RT were well tolerated with no acute Grade 3 toxicity. There were more documented cases of Grade 2 fatigue occurring in the second half of chemo-RT with the 6-week CF-RT schedule compared to the 4-week HF-RT schedule. While it is tempting to view this as corroborative of Level I data in the breast cancer setting which also showed less fatigue with HF-RT [37], no strong conclusions can be drawn here given the small numbers and retrospective nature of this study. The higher rate of radionecrosis in the HF-RT group may be related to the higher proportion of tumour MGMT methylation in the group [38]. Interestingly, Mallick et al. and Navarria et al. also reported higher radionecrosis rates with HF-RT compared to CF-RT [25, 30]. Given that vascular injury is a significant mechanism of indirect tumour cell kill in severe hypofractionation (> 10 Gy/fraction) (in contrast to direct tumour cell kill as the major event in CF-RT [39]), we cannot rule out a similar differential also at play with moderate HF-RT, considering that increased tumour and endothelial cell damage are postulated as mechanisms of radionecrosis [40].

Our systematic review of GBM HF-RT literature revealed a heterogenous selection of studies in patient population, trial design and outcomes. To focus our review, we included only studies that had a majority of younger and good performance status patients, and excluded studies that used severe HF-RT such as stereotactic radiosurgery boosts. There is no indication from the literature of poorer survival using HF-RT. The pooled OS and PFS for HF-RT with temozolomide compare favourably with that of the landmark EORTC/NCIC trial [3] (17.5 and 9.9 months, versus 14.6 and 6.9 months, respectively). Focusing only on trials that used HF-RT to reduce overall treatment time (54-60 Gy in 15–20 fractions with temozolomide), these also demonstrated outcomes that are at least similar to, if not potentially better than, that of CF-RT, with median OS of 15.0–25.2 months [25, 26, 28, 30, 41]. Toxicity is poorly described, but Grade ≥ 3 neurological toxicity and radionecrosis were largely seen in studies that used a simultaneous-integrated boost of ≥ 70 Gy total on a CF-RT backbone to a larger volume, at a rate of up to 29%. This degree of risk was not reflected in studies that used HF-RT to shorten overall treatment time (< 5 weeks), in which the rates of Grade ≥ 3 radionecrosis were 0–2.4%. For comparison, in trials utilising severe hypofractionation (> 5 Gy/fraction), Grade ≥ 3 radionecrosis rates were 16–20% [31, 42–44]. Thus, it appears that the risk of radionecrosis is associated with a very high total dose and/or large fraction size, rather than moderate hypofractionation (< 3 Gy/fraction).

It should be acknowledged that the impact of moderate HF-RT on late normal tissue complications in the brain, especially for the rare proportion of long-term GBM survivors, is difficult to assess and at present unknown. On the flipside, shorter courses of daily RT treatments can offer a significant reduction in patient and carer burden, especially for this devastating disease. Another advantage of shorter HF-RT schedules for the GBM patient is the increased likelihood of treatment completion. In two Phase 3 studies comparing HF-RT with CF-RT in elderly GBM patients (> 60–65 years), patients receiving HF-RT were substantially less likely to abandon treatment due to clinical deterioration or disease progression, compared to those receiving CF-RT [9, 10]. This observation is corroborated in the young, fit patient subgroup by the propensity-matched comparison by Navarria et al., as described above [30]. On a larger-scale, the positive impact on healthcare costs and waiting lists from adopting HF-RT in other cancer types such as breast and prostate cancers are well discussed in the literature [45]. Looking forward, the challenges imposed by the COVID-19 pandemic have been instructive on the potential advantages of HF-RT in a resource-scarce environment and for patient treatment convenience, serving as a catalyst for more studies to investigate the adoption of shorter schedules where appropriate in the modern RT era [46, 47].

## Conclusion

The COVID-19 pandemic has necessitated pragmatic changes in cancer management including for GBM. In this context, there appears to be comparable outcomes for young and fit GBM patients using a 4-week HF-RT course compared to 6 weeks of CF-RT, with no undue toxicity. This is supported by a systematic review and meta-analysis of HF-RT studies in this patient subgroup. Given the emerging data supporting the use of a shorter HF-RT course for these patients, as well as its potential patient, carer, and healthcare system benefits, larger studies should investigate this as the next evolution in the RT management of GBM patients.

## Electronic supplementary material

Below is the link to the electronic supplementary material.

Supplementary Material 1

## References

[1] Gan HK, Rosenthal MA, Cher L, Dally M, Drummond K, Murphy M (2015). Management of glioblastoma in Victoria, Australia (2006–2008). J Clin neuroscience: official J Neurosurgical Soc Australasia.

[2] Bleehen NM, Stenning SP (1991). A Medical Research Council trial of two radiotherapy doses in the treatment of grades 3 and 4 astrocytoma. The Medical Research Council Brain Tumour Working Party. Br J Cancer.

[3] Stupp R, Mason WP, van den Bent MJ, Weller M, Fisher B, Taphoorn MJ (2005). Radiotherapy plus concomitant and adjuvant temozolomide for glioblastoma. N Engl J Med.

[4] Gondi V, Pugh S, Tsien C, Chenevert T, Gilbert M (2020). Radiotherapy (RT) dose-intensification (DI) using intensity-modulated RT (IMRT) versus standard-dose (SD) RT with temozolomide (TMZ) in newly diagnosed glioblastoma (GBM): preliminary results of NRG Oncology BN001". Int J Radiat Oncol Biol Phys.

[5] Gilbert MR, Wang M, Aldape KD, Stupp R, Hegi ME (2013). Dose-dense temozolomide for newly diagnosed glioblastoma: a randomized phase III clinical trial. J Clin Oncol.

[6] Chinot OL, Wick W, Mason W, Henriksson R, Saran F (2014). Bevacizumab plus radiotherapy–temozolomide for newly diagnosed glioblastoma. New Eng J Med.

[7] Stupp R, Hegi ME, Gorlia T, Erridge SC, Perry J (2014). Cilengitide combined with standard treatment for patients with newly diagnosed glioblastoma with methylated MGMT promoter (CENTRIC EORTC 26071 – 22072 study): a multicentre, randomised, open-label, phase 3 trial. Lancet oncol.

[8] Qi XS, Schultz CJ, Li XA (2006). An estimation of radiobiologic parameters from clinical outcomes for radiation treatment planning of brain tumor. Int J Radiat Oncol Biol Phys.

[9] Malmström A, Grønberg BH, Marosi C, Stupp R, Frappaz D, Schultz H (2012). Temozolomide versus standard 6-week radiotherapy versus hypofractionated radiotherapy in patients older than 60 years with glioblastoma: the Nordic randomised, phase 3 trial. Lancet Oncol.

[10] Roa W, Brasher PM, Bauman G, Anthes M, Bruera E, Chan A (2004). Abbreviated course of radiation therapy in older patients with glioblastoma multiforme: a prospective randomized clinical trial. J Clin Oncol.

[11] Perry JR, Laperriere N, O’Callaghan CJ, Brandes AA, Menten J, Phillips C (2017). Short-Course Radiation plus Temozolomide in Elderly Patients with Glioblastoma. N Engl J Med.

[12] Mohile NA, Blakeley JO, Gatson NTN, Hottinger AF, Lassman AB (2020). Urgent Considerations for the Neuro-oncologic Treatment of Patients with Gliomas During the COVID-19 Pandemic. Neuro Oncol.

[13] Kochbati L, Vanderpuye V, Moujahed R, Rejeb MB, Naimi Z, Olasinde T (2020). Cancer care and COVID-19: tailoring recommendations for the African radiation oncology context. Ecancermedicalscience.

[14] Weller M, Preusser M (2019). How we treat patients with brain tumour during the COVID-19 pandemic. ESMO open.

[15] Airth A, Whittle JR, Dimou J (2022). How has the COVID-19 pandemic impacted clinical care and research in neuro-oncology?. J Clin Neurosci.

[16] Louis DN, Perry A, Wesseling P, Brat DJ, Cree IA (2021). The 2021 WHO Classification of Tumors of the Central Nervous System: a summary. Neuro Oncol.

[17] Ly KI, Gerstner ER (2018). The Role of Advanced Brain Tumor Imaging in the Care of Patients with Central Nervous System Malignancies. Curr Treat Options Oncol.

[18] Niyazi M, Brada M, Chalmers AJ, Combs SE, Erridge SC (2016). ESTRO-ACROP guideline “target delineation of glioblastomas". Radiother Oncol.

[19] Bell EH, Pugh SL, McElroy JP, Gilbert MR, Mehta M (2017). Molecular-Based Recursive Partitioning Analysis Model for Glioblastoma in the Temozolomide Era: A Correlative Analysis Based on NRG Oncology RTOG 0525. JAMA Oncol.

[20] DerSimonian R, Laird N (2015). Meta-analysis in clinical trials revisited. Contemp Clin Trials.

[21] Massaccesi M, Ferro M, Cilla S, Balducci M, Deodato F (2013). Accelerated intensity-modulated radiotherapy plus temozolomide in patients with glioblastoma: a phase I dose-escalation study (ISIDE-BT-1). Int J Clin Oncol.

[22] Monzajeb A, Ayala D, Jensen C, Case L, Bourland J (2012). A Phase I Dose Escalation Study of Hypofractionated IMRT Field-in-Field Boost for Newly Diagnosed Glioblastoma Multiforme. Int J Radiat Oncol Biol Phys.

[23] Tsien C, Brown D, Normolle D, Schipper M, Piert M (2012). Concurrent temozolomide and dose-escalated intensity-modulated radiation therapy in newly diagnosed glioblastoma.

[24] Jastaniyah N, Murtha A, Pervez N, Le D, Roa W (2013). Phase I study of hypofractionated intensity modulated radiation therapy with concurrent and adjuvant temozolomide in patients with glioblastoma multiforme. Radiat Oncol.

[25] Mallick S, Kunhiparambath H, Gupta S, Benson R, Sharma S (2018). Hypofractionated accelerated radiotherapy (HART) with concurrent and adjuvant temozolomide in newly diagnosed glioblastoma: a phase II randomized trial (HART-GBM trial). J Neuro Oncol.

[26] Scoccianti S, Krengli M, Marrazzo L, Magrini S, Detti B (2018). Hypofractionated radiotherapy with simultaneous integrated boost (SIB) plus temozolomide in good prognosis patients with glioblastoma: a multicenter phase II study by the Brain Study Group of the Italian Association of Radiation Oncology (AIRO). Radiol med.

[27] Patel S, Amanie J, Murtha A, Rowe L, Easaw J (2021). A randomized trial of short-course versus conventional radiotherapy with concomitant and adjuvant temozolomide in patients 18 to 70 years of age with glioblastoma. Int J Radiation Oncol Biol Phys.

[28] Azoulay M, Santos F, Souhami L, Panet-Raymond V, Petrecca K (2015). Comparison of radiation regimens in the treatment of Glioblastoma multiforme: results from a single institution. Radiat Oncol.

[29] Guler O, Yildirim B, Onal C, Topkan E (2019). Retrospective comparison of standard and escalated dose of radiotherapy in newly diagnosed glioblastoma patients tretaed with concurrent and adjuvant temozolomide. Indian J Cancer.

[30] Navarria P, Pessina F, Franzese C, Tomatis S, Perrino M (2018). Hypofractionated radiation therapy (HFRT) versus conventional fractionated radiation therapy (CRT) for newly diagnosed glioblastoma patients. A propensity score matched analysis. Radiother and Oncol.

[31] Floyd N, Woo S, Teh B, Prado C, Mai W-Y (2004). Hypofractionated Intensity-Modulated Raditotherapy for Primary Glioblastoma Multiforme. Int J Radiat Oncol Biol Phys.

[32] Phillips C, Guiney M, Smith J, Hughes P, Narayan K, Quong G (2003). A radomised trial comparing 35Gy in ten fractions with 60 Gy in 30 fractions of cerebral irradiation for glioblastoma multiforme and older patients with anaplastic astrocytoma. Radio Oncol.

[33] Sultanem K, Patrocinio H, Lambert C, Corns R, Leblanc R (2004). The Use of Hypofractionated intensity-modulated irradiation in the treatment of glioblastoma multiforme: preliminary results of a prospective trial. Int J Radiat Oncol Biol Phys.

[34] Usman S, Chaudry S, Hameed S, Hussain K, Butt S (2015). Hypofractionated Radiotherapy in Glioblastoma Multiforme. J Cancer Allied Spec.

[35] Zhong L, Chen L, Lv S, Li Q, Chen G (2019). Efficacy of moderately hypofractionated simultaneous integrated boost intensity modulated radiotherapy combined with temozolomide for the postoperative treatment of glioblastoma multiforme: a single-institution experience. Radiat Oncol.

[36] Zschaeck S, Wust P, Graf R, Misch M, Onken J, Ghadjar P (2018). Locally dose-escalated radiotherapy may improve intracranial local control and overall survival among patients with glioblastoma. Radiat Oncol.

[37] Shaitelman SF, Schlembach PJ, Arzu I, Ballo M, Bloom E (2015). Acute and Short-term Toxic Effects of Conventionally Fractionated vs Hypofractionated Whole-Breast Irradiation: A Randomized Clinical Trial. JAMA Oncol.

[38] Brandes AA, Franceschi E, Tosoni A, Blatt V, Pession A (2008). MGMT promoter methylation status can predict the incidence and outcome of pseudoprogression after concomitant radiochemotherapy in newly diagnosed glioblastoma patients. J Clin Oncol.

[39] Song CW, Glatstein E, Marks LB, Emami B, Grimm J (2021). Biological Principles of Stereotactic Body Radiation Therapy (SBRT) and Stereotactic Radiation Surgery (SRS): Indirect Cell Death. Int J Radiat Oncol Biol Phys.

[40] Brandsma D, Stalpers L, Taal W, Sminia P, van den Bent MJ (2008). Clinical features, mechanisms, and management of pseudoprogression in malignant gliomas. Lancet Oncol.

[41] Shenouda G, Souhami L, Petrecca K, Owen S, Panet-Raymod V (2017). A Phase 2 Trial of Neoadjuvant Temozolomide Followed by Hypofractionated Accelerated Radiation Therapy With Concurrent and Adjuvant Temozolomide for Patients With Glioblastoma. Int J Radiation Oncol Biol Phys.

[42] Miwa K, Matsuo M, Ogawa S, Shinoda J, Asano Y (2014). Hypofractionated high-dose irradiation with positron emission tomography data for the treatment of glioblastoma multiforme. Biomed Res Int.

[43] Reddy K, Damek D, Gaspar L, Ney D, Waziri A (2012). Phase II Trial of Hypofractionated IMRT with temozolomide for patients with newly diagnosed glioblastoma multiforme. Int J Radiat Oncol Biol Phys.

[44] Yoon S, Kim J, Kim S, Khang S, Shin S (2013). Hypofractionated intensity-modulated radiotherapy using simultaneous integrated boost technique with concurrent and adjuvant temozolomide for glioblastoma. Tumori.

[45] Hunter D, Mauldon E, Anderson N (2018). Cost-containment in hypofractionated radiation therapy: a literature review. J Med Radiat Sci.

[46] Guckenberger M, Belka C, Bezjak A, Bradley J, Daly ME (2020). Practice recommendations for lung cancer radiotherapy during the COVID-19 pandemic: An ESTRO-ASTRO consensus statement. Radiother and Oncol.

[47] Thomson DJ, Palma D, Guckenberger M, Balermpas P, Beitler JJ (2020). Practice recommendations for risk-adapted head and neck cancer radiation therapy during the COVID-19 pandemic: an ASTRO-ESTRO consensus statement. Int J Radiat Oncol Biol Phys.

