# Supplementary material for: Moderately hypofractionated versus conventionally fractionated radiation therapy with temozolomide for young and fit patients with glioblastoma: an institutional experience and meta-analysis of literature
Source: J Neurooncol. 2022 Nov 10;160(2):361–74. doi: 10.1007/s11060-022-04151-z (PMC9648463; doi:10.1007/s11060-022-04151-z)
Supplement: Supplementary file 1 — Supplementary Material 1 [file 11060_2022_4151_MOESM1_ESM.docx]

**Title: Moderately Hypofractionated versus Conventionally Fractionated Radiation Therapy with Temozolomide for Young and Fit Patients with Glioblastoma: An Institutional Experience and Meta-analysis of Literature**

**Journal:** Journal of Neuro-Oncology

**Authors:** Phoebe Chidley**,** Mihir Shanker, Claire Phillips, Neda Haghighi, Mark B Pinkham, James R Whittle, Joseph Sia

**Corresponding Author:**

Joseph Sia

Department of Radiation Oncology

Peter MacCallum Cancer Centre, Melbourne, 3000, Australia

E-mail: [joseph.sia@petermac.org](mailto:joseph.sia@petermac.org)

**Supplementary Table 1. Other hypofractionation treatment studies for Glioblastoma**

| **Reference** | **Study design** | ***n*** | **Patient characteristics** | | **Arms** | | | **Outcomes** | | **Toxicity** |
| --- | --- | --- | --- | --- | --- | --- | --- | --- | --- | --- |
|  |  |  | **Median age (years)** | **Performance Status** | **Dose Fractionation** | **Dose Levels** | **TMZ Use** | **Median OS (months)** | **Median PFS (months)** |  |
| **Arslan 2006^a^** | Prospective | 20 | 57 (range 37-86) | ECOG 3-4 in 47.6% | 50Gy/15# over 5 weeks | Two | No | 13.5 | 6.8 | 1 patient (severe) RN |
|  |  |  | ND | ECOG 3-4 in 64.7% | Historical control 60Gy/30# | ND | No | 11.3 (*P* = 0.16) | 5.6 (*P =*0.36) |  |
| **Arvold 2015^b^** | Retrospective | 135 | 79 | Median KPS 70 | 40.05Gy/15# | Two | Yes – 79% | RT alone 4.1 (range 1.8-12.8);  RT with TMZ 9.6 (2.7-75.8) | RT alone 2.2 (range 1.2-4.8);  RT with TMZ 4.5 (1-73) | ND |
|  |  |  | 69 | Median KPS 80 | Std # (59.4Gy/33# or 60Gy/30#) | Two | Yes – 62.9% | RT alone 9.5 (2.5-41.7); RT with TMZ 11.0 (3.4-45.3) | RT alone 6.0 (0.4-30.8); RT with TMZ 6.1 (1.5-30.4) | ND |
| **Bracci 2016^c^** | Retrospective | 21 | 82 (range 80-88) | Median KPS 80 | 40Gy/15# | One | Yes – 47.6% | 7.5 (95% CI 4.5 - 19.1) | 5.8 (95% CI 3.9-7.7) | 14.3% pts hospitalised during RT, 38.1% required commencement/ increase to steroid dosing |
| **Choudhary 2019^d^** | Prospective randomised | 40 | 54.4 | KPS 30-80: 75% | 35Gy/7# (over 15 days) | One | No | 7.2 | 5.5 | No significant difference in neurological performance scale (*P* =0.31) |
|  |  |  | 46 | KPS 30-80: 85% | 60Gy/30# | One | No | 10.4 (*P* = 0.09) | 3.8 (*P* =0.34) |  |
| **Chen 2011^e^** | Prospective Phase I | 16 | 69 (range 34-84) | Median KPS 80 | Dose escalation PTV 60Gy/20# vs 60Gy/15# vs 60Gy/12# vs 60Gy/10# | Two | Yes | 16.2 (range 2-32.6) | ND | No dose-limiting toxicity seen.  Late: 1 x ipsilateral vision loss; 3 x RN |
| **Ciammella 2013^f^** | Retrospective Case series | 67 | 64.5 (range 41-82) | Median KPS 80 | 25Gy/5# (prescribed to 70% isodose) | One | Yes - adj | 13.4 (95% CI 8.21-16.4) | 7.9 (95% CI 6.6-8.3) | No Gr3-4 acute tox observed. Gr3 late tox (haem) in 10 pts. |
| **Greer 2017^g^** | Retrospective | 20 | 70.5 (61-82) | Median KPS 60 (40-90) | 36Gy/6# (alternate days) | Two | Yes | 14.0 (95% CI: 5.0, not estimable) | 5.0 (95% CI: 2.8, 16.4) | 2 pts did not complete RT. No acute Gr 3+ toxicity in those that completed RT. |
| **Gregucci 2021^h^** | Retrospective | 25 | 65 | Median KPS 60 | 40.05Gy/15# with SIB 52.5Gy to cavity/ residual/ gross disease | Two | Yes | 13.0 (95% CI 9.8-na) | 8.4 (95% CI 5.8-11.9%) | No Gr2+ neuro tox reported, 3 cases Gr3-4 haem tox |
| **Guedes de Castro 2017^i^** | Post hoc subset analysis (of IAEA phase 3 RCT) | 61 | Eligibility: Age > 65y | Majority in both arms KPS 60-80% | 25Gy/5# | One | No | 6.8 (95% CI 4.5-9.1) | 4.3 (95% CI 2.6-5.9) | No acute Gr3+ tox. |
|  |  |  |  |  | 40Gy/15# | One | No | 6.2 (95% CI 4.7-7.7) [*P* = 0.936] | 3.2 (95% CI 0.1- 6.3) [P = 0.706] |  |
| **Hulshof 2000^j^** | Prospective | 155 | Mean ages 54-60 | MRC neuro PS 3-4 (major functional impairment to no useful function) in 36%. Other PS not described. | 66Gy/33# | ND | No | 7.0 | ND | No difference in acute toxicity. Late toxicity ND. |
|  |  |  |  |  | 40Gy/8# | ND | No | 5.6 | ND |  |
|  |  |  |  |  | 28Gy/4# | ND | No | 6.6 | ND |  |
| **Iuchi 2013^k^** | Prospective Phase 2 trial | 46 | 65.5 (range 40-80) | RPA Class V or VI | 3 dose levels 68Gy, 40Gy and 32Gy in 8# | Three | Yes | 20.0  2yOS 42.8% | -  2y local PFS 63.9% | 20 pts RN, ungraded |
| **Jablonska 2019^l^** | Prospective | 17 | 68 (range 50-77) | Median KPS 50-70% | 3 dose levels 50Gy, 45Gy, 40Gy in 15# | Three | Yes | 7.0 | 7.0 (95% CI 5.7-8.2) | 1 case Gr3 RN |
| **Lee 2021^m^** | Retrospective | 62 | 74 (range 70-90) | KPS >70: 74% | Std 60Gy/30# | Two | Yes | 12.4 (95% CI 9-16.4) | ND | ND |
|  |  |  |  | KPS >70: 63% | 40.05Gy/15# | One | Yes | 9.9 (95% CI 3.4-15.1) | ND | ND |
| **Lombardi 2015^n^** | Retrospective, multi-centre | 237 | 71 (range 65-84) | ECOG 0-1 in 83% pts | Std 60Gy/30# | One | Yes | 19.4 (95% CI 16.3-21.5) | ND | Haem Gr 3-4 acute tox in 10% patients.  Non-haem tox potentially related to RT: 2 patients with asthenia, 1 with nausea. |
|  |  |  |  |  | 40Gy/15# | One | Yes | 13.8 (95% CI 11.7-17.9) [*P*= 0.02] | ND |  |
| **Malmstrom 2012^o^** | Prospective Phase 3 randomised | 291 | 70 | ECOG 0-1: 78% | TMZ | - | Yes | 8.3 [95% CI 7.1-9.5] | ND | No RN recorded |
|  |  |  |  | ECOG 0-1: 80% | 34Gy/10# | ND | No | 7.5 [6.5-8.6] | ND |  |
|  |  |  |  | ECOG 0-1: 72% | 60Gy/30# | ND | No | 6.0 [95% CI 5.1-6.8] | ND |  |
| **Minniti 2009^p^** | Prospective | 43 | 73 (range 70-79) | Median KPS 70 | 30Gy/6# | One | Yes - adj | 9.3 (95% CI 7.5-11.1) | 6.3 (95% CI 4.8-7.8) | 28% Gr3-4 haem tox. Neuro deterioration during/post RT in 16%, majority resolved with steroid use. |
| **Minniti 2012^q^** | Prospective Phase II multi-centre | 71 | 73 (range 70-81) | Median KPS 70 | 40Gy/15# | One | Yes | 12.4 (95% CI 9.9-15)  2yOS 20% (95% CI 6-38%) | 2yPFS 5% (95% CI 1-12%) | Gr3-4 tox 22% (15% Gr3-4 haem tox). Non-haem: Acute Gr 2 confusion 4 pts (reversible with steroids), Gr3 Fatigue 4 pts, Cognitive disability 1 pt. Late Gr2 neurologic deterioration 3 pts; Radiologic leukoencephalopathy seen in 19 (of 39 pts) alive at 12 months. |
| **Minniti 2014^r^** | Retrospective propensity-matched analysis | 243 | 68 | Median KPS 70 | 60Gy/30# | One | Yes | 12.0  2yOS: 18% (95% CI 5-31%) | 5.6 | 8.6% did not complete RT due to deterioration.  Late neurologic deterioration 12 pts (without evidence recurrence). Overall 40% Gr2-3 neuro tox |
|  |  |  | 71 | Median KPS 70 | 40Gy/15# | One | Yes | 12.5 (p=0.5).  2yOS: 12% (95% CI 3-21%) | 6.7 | 1.7% did not complete RT due to deterioration.  Late neurologic deterioration 3 pts (without evidence recurrence).  Overall 14% Gr2-3 neuro tox |
| **Miwa 2014^s^** | Prospective | 45 | 61 (range 21-86) | KPS > 70 in 68.9% | 68Gy/8# to region of MET-PET uptake (56Gy and 40Gy to CTV and PTV respectively) | Two | Yes | 20.0  2yOS 26.3% | 13.0  2yPFS 20.6% | Non-Haem Acute: Gr1-2 nausea/vomiting 8.8%). Nil Gr3-4.  Late: RN Gr3 11.1%, Gr4 4.4%; Haemorrhage Gr3 2.2%, Gr4 2.2%; Cerebropathy Gr3 2.2% |
| **Muni 2010^t^** | Prospective | 45 | 66 | Median KPS 70 | 30Gy/6# (over 2 weeks) | One | No | 7.3 | 4.4 | Haem tox Gr3-4 in TMZ arm.  Neuro: 2 pts did not complete RT due to neurologic deterioration. 9 pts with Gr2-3 confusion and/or somnolence, 6 of these had reversal symptoms with steroid use |
|  |  |  | 67 |  | 30Gy/6# (over 2 weeks) | One | Yes - adj | 9.4 (*P* = 0.003) | 5.5 (P = 0.03) |  |
| **Navarria 2019^u^** | Prospective single-arm Phase II | 30 | 75 (range 71-83) | KPS 60 for 86.7% pts | 52.5Gy/15# | One | Yes – 64%, adj +/- conc | 8.0 (95% CI 7.1-10.4) | 5.0(95% CI 4.1-7.3) | Gr2 Haem tox in 1 TMZ pt. Gr2-3 fatigue in 3 pts. 90% of pts did not have severe acute or late neurologic toxicity recorded. |
| **Panet-Raymond 2009^v^** | Retrospective | 35 | 63 (range 31-78) | RTOG RPA Class: III in 5.7%, IV in 20%, V in 57.1% and VI in 17.1% | Single arm using concomitant boost (60Gy/20#) | Two | Yes | 14.4 | 7.7 | 82.8% pts completed concurrent treatment.  1 pt Gr3-4 tox (vomiting) during adj TMZ |
| **Perry 2017^w^** | Prospective randomised Phase 3 | 562 | 73 (range 65-90) | ECOG 0-1: 76.9% | 40Gy/15# | One | No | 7.6 | 3.9 | Similar QoL both groups.  Higher haem tox with addition of TMZ. 2 deaths in each group attributed to treatment. |
|  |  |  |  |  | 40Gy/15# | One | Yes | 9.3 [HR for death, 0.67; 95% CI, 0.56-0.8; p<0.001)] | 5.3 [HR for progression or death, 0.5; 95%CI, 0.41-0.6; p<0.001)] |  |
| **Rayan 2020^x^** | Prospective, single institution | 93 | 55 | KPS < 70: 41.7% | 45Gy/15# | Two | Yes - conc | 9.0 (95% CI: 7.91-10.09) | 6.0 (95% CI: 5.2-6.8) | No significant difference seen between arms |
|  |  |  | 50 | KPS < 70: 41.4% | 60Gy/30# | Two | Yes - conc | 11.0 (95%CI: 8.19-13.8, p=0.374) | 10.0 (95%CI: 6.77-13.2, *p*=0.087) |  |
| **Reddy 2012^y^** | Prospective Phase 2 trial | 24 | 60.5 (range 27-77) | Median KPS 80 | Concomitant boost to 60Gy/10# (lower dose 30Gy/10#) | Two | Yes | 16.6 (range 4.1-35.9mos) | ND | With median follow-up of 14.8mos, no Gr3+ acute or late non-haem tox observed. Re-do craniotomy in 6 pts (2 symptomatic), with 4 of these with histo-confirmed RN |
| **Roa 2004^z^** | Prospective randomised | 100 | 71 | Median KPS 70 | 60Gy/30# | Two | No | 5.1 | ND | 49% corticosteroid requirement |
|  |  |  | 72.4 | Median KPS 70 | 40Gy/15# | Two | No | 5.6 (HR 0.89; 95%CI: 0.59-1.36; p=0.57) | ND | 23% corticosteroid requirement |
| **Roa 2015^aa^** | Phase 3 randomised | 98 | 54% >65 | Mean KPS 64 | 25Gy/5# | One | No | 7.9 (95%CI: 6.3-9.6) | 4.2 (95%CI: 2.5-5.9) | No acute Gr3+ tox. No diff corticosteroid use between arms.  No SS diff QoL between arms at 4 and 8wks post RT. |
|  |  |  | 70% >65 | Mean KPS 65.8 | 40Gy/15# | One | No | 6.4 (95%CI: 5.1-7.6) (*P*=0.988) | 4.2 (95%CI: 2.6-5.7) (P=0.716) |  |
| **Shenouda 2016^bb^** | Prospective Phase 2 trial | 50 | 60 (range 31-79y) | Median ND | Neo-adj TMZ (started 2-3 wks post op) followed by 60Gy/20# | Two | Yes – including neo-adj | 22.3 (95%CI: 14.6-42.7)  4yOS 30.4% (MGMT methylated 53.3% vs unmethylated 14.0%) | 13.7 (95%CI: 8.0-33.3) | 1 Gr5 pancytopaenia, 1 transient Gr4 hepato-tox. 13 pts required 2nd surg procedure (2 infection, 3 recurrences, 4 recurrences + RT-induced damage, 4 RT-induced damage alone) |
| **Terasaki 2011^cc^** | Prospective, pilot study | 26 | 61 (39-79) | Median KPS 60 | 45Gy/15# | One | Yes | 15.6 (95% CI 9-22) | 9.6 (95% CI 6.3-12.9) | Nil RN observed |
| **Wang 2016^dd^** | Retrospective | 158 | 72 | 73.1% KPS >70 | 40Gy/15# | Two | Yes | 15.6 |  | ND |
|  |  |  | 66 | 82.6% KPS >70 | Std # (60Gy/3# or 59.4Gy/33#) | Two | Yes | 14.1 (*P* = 0.550) |  | ND |
| **Wee 2020^ee^** | Retrospective, multi-centre | 260 | Mean 74 (+5.2) |  | 45Gy/15# (IQR 42.5-45Gy) |  |  | > 65y group:  13.2  >70y group: 13.3 |  | ND |
|  |  |  | Mean 69.4y (+3.4) |  | Std 60Gy/33# (IQR 60-61.2Gy) |  |  | > 65y group: 17.6 (*P* <0.001)  >70y group:  16.4 (P = 0.002) |  | ND |
| **Yoon 2013^ff^** | Retrospective | 39 | 55 (range 19-72) | 94.9% RPA Class IV and V. KPS not stated | SIB technique to total 50Gy/10# | Two | Yes | 16.8;  2yOS 38.5% | 6.8 | RN in 7 pts (17.9%) - 3 pts requiring reoperation, with 1 pt (2.6%) experiencing severe necrosis requiring emergency surgery |

Key:

ND = Not described; RN = Radionecrosis; Pt = patient; Gy = Gray; # = fractions; ; y = years; Std = Standard; SIB = Simulated integrated boost; TMZ = Temozolomide; Adj = adjuvant; Conc = Concurrent; KPS = Karnofsky Performance Status; RPA = Recursive Partitioning Analysis; Gr = Grade; tox = Toxicity; QoL = Quality of Life

**Supplementary Table 1 References:**

a. Arslan M, Karadeniz AN, Aksu G, Güveli M, Fayda M, et al. Postoperative hypofractionated radiotherapy in glioblastoma multiforme. J BU ON. 2006;11(1):39-42.

b. Arvold ND, Tanguturi SK, Aizer AA, Wen PY, Reardon DA, et al. Hypofractionated versus standard radiation therapy with or without temozolomide for older glioblastoma patients. Int J Radiat Oncol Biol Phys. 2015;92(2):384-9.

c. Bracci S, Laigle-Donadey F, Hitchcock K, Duran-Peña A, Navarro S, et al. Role of irradiation for patients over 80 years old with glioblastoma: a retrospective cohort study. J Neuro-Oncol. 2016;129(2):347-53.

d. Choudhary S, Gupta N, Lal P, Kumar S. Short Course Hypofractionated Radiotherapy versus Conventional Radiotherapy for Poor Prognosis Glioblastoma Multiforme: A Randomised Controlled Trial. J Clin Diag Res. 2019;13(3).

e. Chen C, Damek D, Gaspar LE, Waziri A, Lillehei K, et al. Phase I trial of hypofractionated intensity-modulated radiotherapy with temozolomide chemotherapy for patients with newly diagnosed glioblastoma multiforme. Int J Radiat Oncol Biol Phys. 2011;81(4):1066-74.

f. Ciammella P, Galeandro M, D’Abbiero N, Podgornii A, Pisanello A, et al. Hypo-fractionated IMRT for patients with newly diagnosed glioblastoma multiforme: a 6 year single institutional experience. Clin Neurol Neurosurg. 2013;115(9):1609-14.

g. Greer L, Pannullo SC, Smith AW, Taube S, Yondorf MZ, et al. Accelerated hypofractionated radiotherapy in the era of concurrent temozolomide chemotherapy in elderly patients with glioblastoma multiforme. Cureus. 2017;9(6).

h. Gregucci F, Surgo A, Bonaparte I, Laera L, Ciliberti MP, et al. Poor-prognosis patients affected by glioblastoma: retrospective study of hypofractionated radiotherapy with simultaneous integrated boost and concurrent/adjuvant temozolomide. J Pers Med. 2021;11(11):1145.

i. de Castro DG, Matiello J, Roa W, Ghosh S, Kepka L, et al. Survival outcomes with short-course radiation therapy in elderly patients with glioblastoma: data from a randomized phase 3 trial. Int J Radiat Oncol Biol Phys. 2017;98(4):931-8.

j. Hulshof MC, Schimmel EC, Bosch DA, González DG. Hypofractionation in glioblastoma multiforme. Radiotherapy and Oncology. 2000;54(2):143-8.

k. Iuchi T, Hatano K, Kodama T, Sakaida T, Yokoi S, et al. Phase 2 trial of hypofractionated high-dose intensity modulated radiation therapy with concurrent and adjuvant temozolomide for newly diagnosed glioblastoma. Int J Radiat Oncol Biol Phys. 2014 Mar 15;88(4):793-800.

l. Jablonska PA, Diez-Valle R, Pérez-Larraya JG, Moreno-Jiménez M, Idoate MÁ, et al. Hypofractionated radiation therapy and temozolomide in patients with glioblastoma and poor prognostic factors. A prospective, single-institution experience. PLoS One. 2019;14(6):e0217881.

m. Lee JW, Kirkpatrick JP, McSherry F, Herndon JE, Lipp ES, et al. Adjuvant Radiation in Older Patients With Glioblastoma: A Retrospective Single Institution Analysis. Front Oncol. 2021;11:631618.

n. Lombardi G, Pace A, Pasqualetti F, Rizzato S, Faedi M, et al. Predictors of survival and effect of short (40 Gy) or standard-course (60 Gy) irradiation plus concomitant temozolomide in elderly patients with glioblastoma: a multicenter retrospective study of AINO (Italian Association of Neuro-Oncology). J Neuro-Oncol. 2015;125(2):359-67.

o. Malmström A, Grønberg BH, Marosi C, Stupp R, Frappaz D, et al. Temozolomide versus standard 6-week radiotherapy versus hypofractionated radiotherapy in patients older than 60 years with glioblastoma: the Nordic randomised, phase 3 trial. Lancet oncol. 2012;13(9):916-26.

p. Minniti G, De Sanctis V, Muni R, Rasio D, Lanzetta G, et al. Hypofractionated radiotherapy followed by adjuvant chemotherapy with temozolomide in elderly patients with glioblastoma. J Neuro-Oncol. 2009;91(1):95-100.

q. Minniti G, Lanzetta G, Scaringi C, Caporello P, Salvati M, et al. Phase II study of short-course radiotherapy plus concomitant and adjuvant temozolomide in elderly patients with glioblastoma. Int J Radiat Oncol Biol Phys. 2012;83(1):93-9.

r. Minniti G, Scaringi C, Lanzetta G, Terrenato I, Esposito V, et al. Standard (60 Gy) or short-course (40 Gy) irradiation plus concomitant and adjuvant temozolomide for elderly patients with glioblastoma: a propensity-matched analysis. Int J Radiat Oncol Biol Phys. 2015;91(1):109-15.

s. Miwa K, Matsuo M, Ogawa SI, Shinoda J, Asano Y, et al. Hypofractionated high-dose irradiation with positron emission tomography data for the treatment of glioblastoma multiforme. BioMed Res Int. 2014;2014.

t. Muni R, Minniti G, Lanzetta G, Caporello P, Frati A, et al. Short-term radiotherapy followed by adjuvant chemotherapy in poor-prognosis patients with glioblastoma. Tumori J. 2010;96(1):60-4.

u. Navarria P, Pessina F, Cozzi L, Tomatis S, Reggiori G, et al. Phase II study of hypofractionated radiation therapy in elderly patients with newly diagnosed glioblastoma with poor prognosis. Tumori J. 2019;105(1):47-54.

v. Panet-Raymond V, Souhami L, Roberge D, Kavan P, Shakibnia L, et al. Accelerated hypofractionated intensity-modulated radiotherapy with concurrent and adjuvant temozolomide for patients with glioblastoma multiforme: a safety and efficacy analysis. Int J Radiat Oncol Biol Phys. 2009;73(2):473-8.

w. Perry JR, Laperriere N, O’Callaghan CJ, Brandes AA, Menten J, et al. Short-course radiation plus temozolomide in elderly patients with glioblastoma. N Eng J Med. 2017;376(11):1027-37.

x. Rayan A, Abdel-Kareem S, Hasan H, Zahran AM, Gamal DA. Hypofractionated radiation therapy with temozolomide versus standard chemoradiation in patients with glioblastoma multiforme (GBM): A prospective, single institution experience. Reports Prac Oncol Radiother. 2020;25(6):890-8.

y. Reddy K, Damek D, Gaspar LE, Ney D, Waziri A, et al. Phase II trial of hypofractionated IMRT with temozolomide for patients with newly diagnosed glioblastoma multiforme. Int J Radiat Oncol Biol Phys. 2012;84(3):655-60.

z. Roa W, Brasher PM, Bauman G, Anthes M, Bruera E, et al. Abbreviated course of radiation therapy in older patients with glioblastoma multiforme: a prospective randomized clinical trial. J Clin Oncol. 2004;22(9):1583-8.

aa. Roa W, Kepka L, Kumar N, Sinaika V, Matiello J, et al. International atomic energy agency randomized phase III study of radiation therapy in elderly and/or frail patients with newly diagnosed glioblastoma multiforme. J Clin Oncol. 2015;33(35):4145-50.

bb. Shenouda G, Souhami L, Petrecca K, Owen S, Panet-Raymond V, et al. A phase 2 trial of neoadjuvant temozolomide followed by hypofractionated accelerated radiation therapy with concurrent and adjuvant temozolomide for patients with glioblastoma. Int J Radiat Oncol Biol Phys. 2017;97(3):487-94.

cc. Terasaki M, Eto T, Nakashima S, Okada Y, Ogo E, Sugita Y, Tokutomi T, Shigemori M. A pilot study of hypofractionated radiation therapy with temozolomide for adults with glioblastoma multiforme. J Neuro-Oncol. 2011;102(2):247-53.

dd. Wang TJ, Wu CC, Jani A, Estrada J, Ung T, et al. Hypofractionated radiation therapy versus standard fractionated radiation therapy with concurrent temozolomide in elderly patients with newly diagnosed glioblastoma. Prac Radiat Oncol. 2016;6(5):306-14.

ee. Wee CW, Kim IH, Park CK, Kim N, Suh CO, et al. Chemoradiation in elderly patients with glioblastoma from the multi-institutional GBM-molRPA cohort: is short-course radiotherapy enough or is it a matter of selection?. J Neuro-Oncol. 2020;148(1):57-65.

ff. Yoon SM, Kim JH, Kim SJ, Khang SK, Shin SS, et al. Hypofractionated intensity-modulated radiotherapy using simultaneous integrated boost technique with concurrent and adjuvant temozolomide for glioblastoma. Tumori J. 2013;99(4):480-7.
